# Supplementary material for: Network Higher-Order Structure Dismantling
Source: Entropy (Basel). 2024 Mar 11;26(3):248. doi: 10.3390/e26030248 (PMC10969674; doi:10.3390/e26030248)
Supplement: Supplementary file 1 [file entropy-26-00248-s001.zip › entropy-2856583-supplementary.pdf]

Supplementary Materials for

Network higher-order structure dismantling

Peng Peng, Tianlong Fan*, Linyuan Lü*

*Corresponding author. Email: tianlong.fan@ustc.edu.cn, linyuan.lv@ustc.edu.cn

1 The performance of BPHD in other parameter conditions

We consider the most extreme case where all higher-order structures are dismantled ($H_k = H_{k-1} = \ldots = H_2 = 0$). If BPHD performs well under this condition, it should perform similarly in other non-extreme scenarios. However, investigating the results of higher-order dismantling under other connectivity targets and non-extreme conditions is indeed necessary. Here, the value of $C = 0.01N$ is widely accepted empirically, we supplement the results for $C = 0.03N$ and $C = 0.06N$, as shown in Figures S1 and S2. Additionally, we consider different higher-order dismantling targets for different orders k. Given the varying initial quantities of structures at different orders, we set $H_k = 0.1H_k^0, 0.2H_k^0, 0.3H_k^0, 0.4H_k^0; C = 0.001N$, and the results are presented in Figures S3-S10. Here, H_k^0 represents the initial number of nodes within k-core.

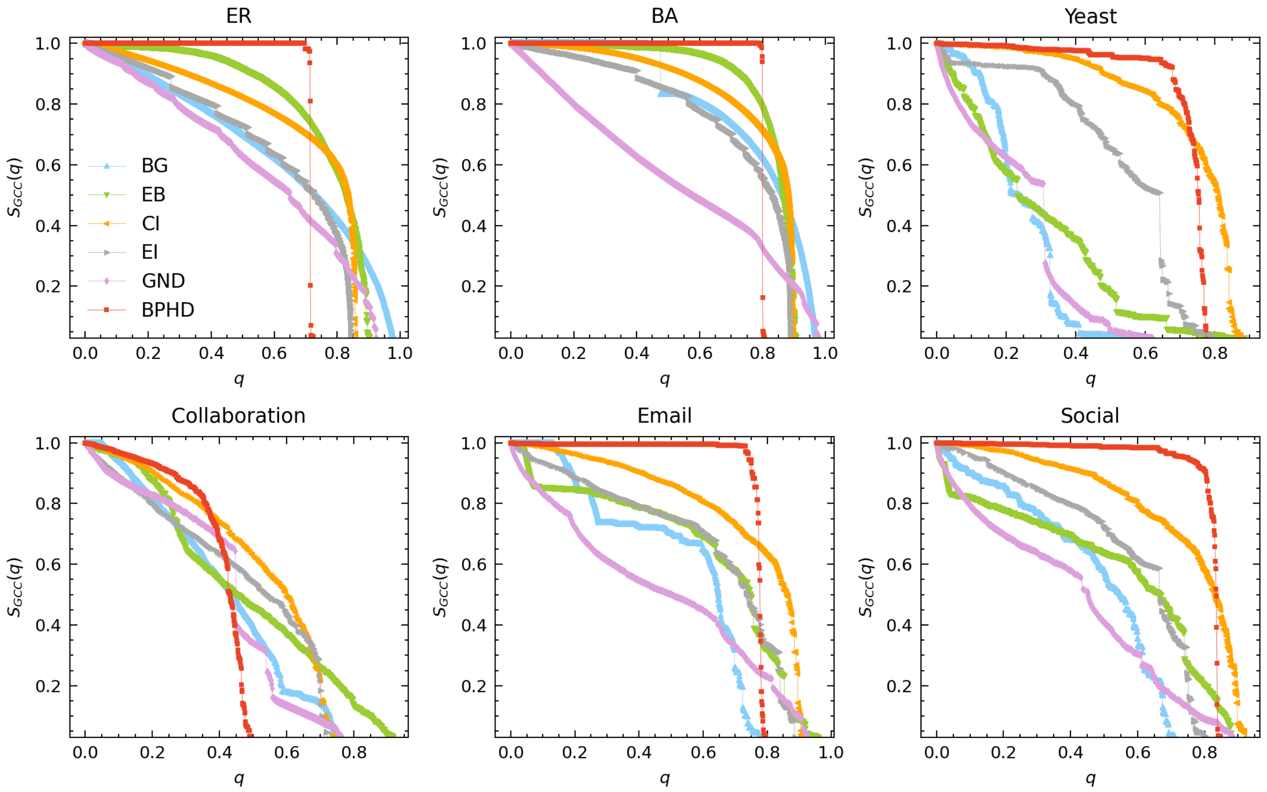

Figure S1: **Performance of BPHD in connectivity dismantling.** The dismantling objectives are set as $C = 0.03N$.

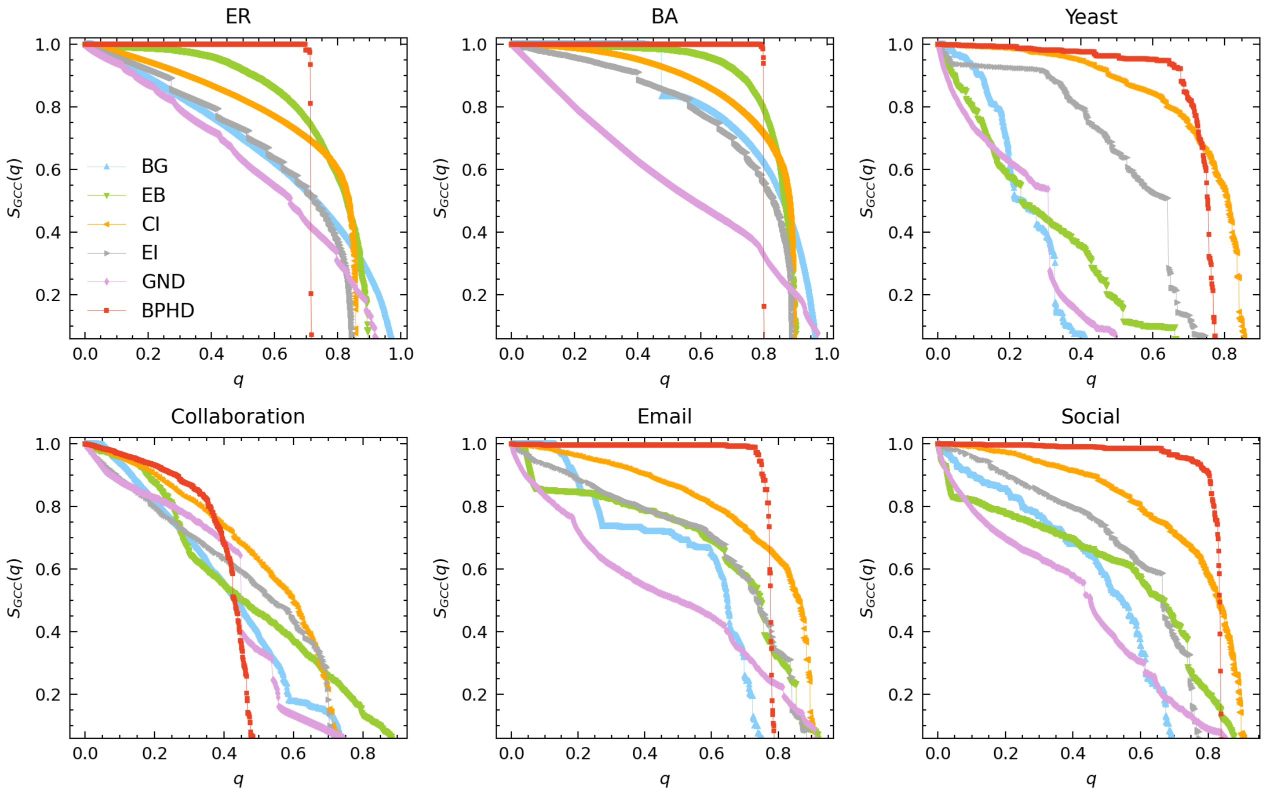

Figure S2: **Performance of BPHD in connectivity dismantling.** The dismantling objectives are set as $C = 0.06N$.

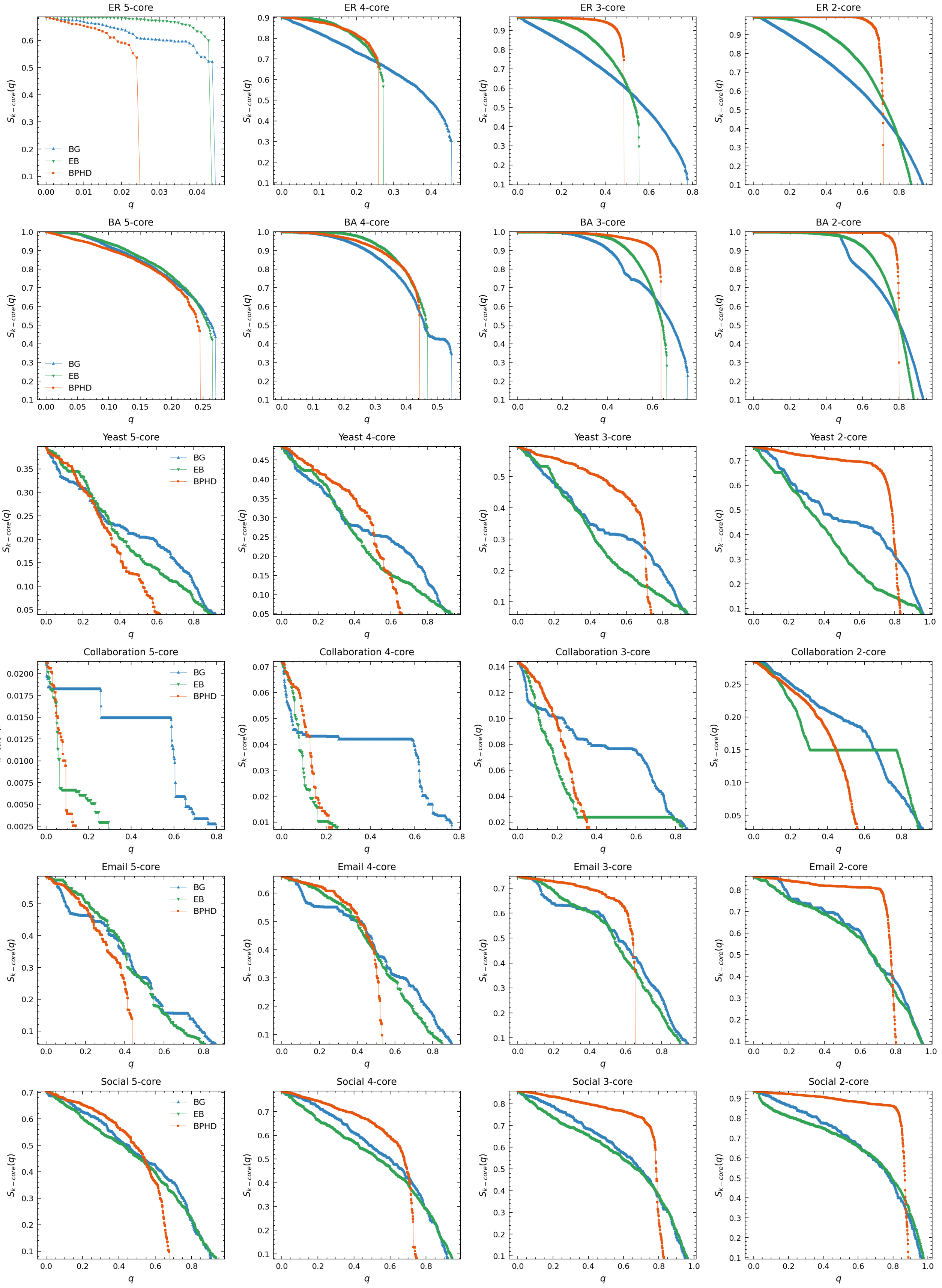

Figure S3: **Performance comparison of BPHD with two classical algorithms in higher-order structure dismantling.** The dismantling objectives are set as $H_k = 0.1H_k^0$, where $k = 5, 4, 3, 2$. H_k^0 represents the initial number of nodes within k-core.

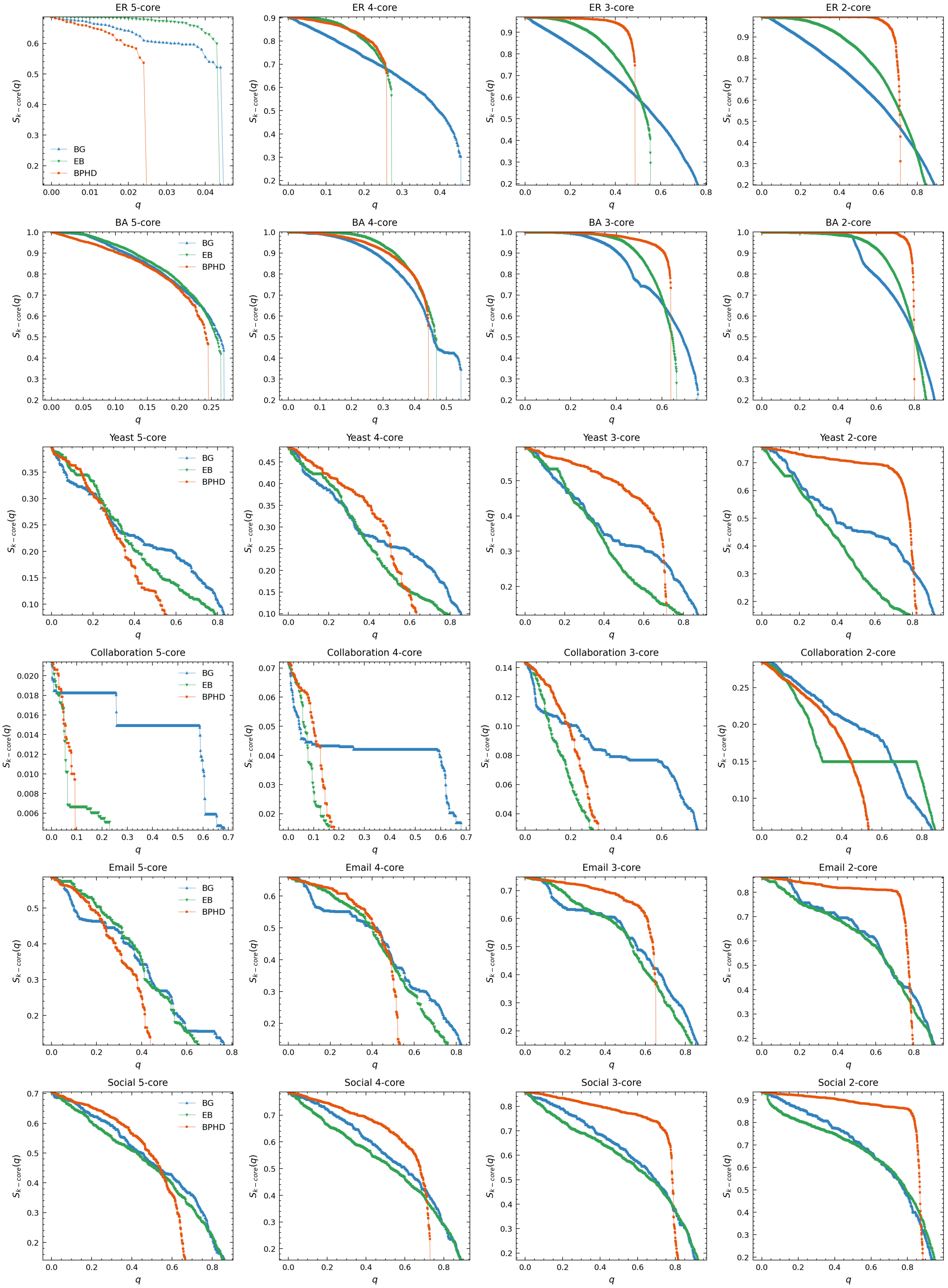

Figure S4: **Performance comparison of BPHD with two classical algorithms in higher-order structure dismantling.** The dismantling objectives are set as $H_k = 0.2H_k^0$, where $k = 5, 4, 3, 2$. H_k^0 represents the initial number of nodes within k-core.

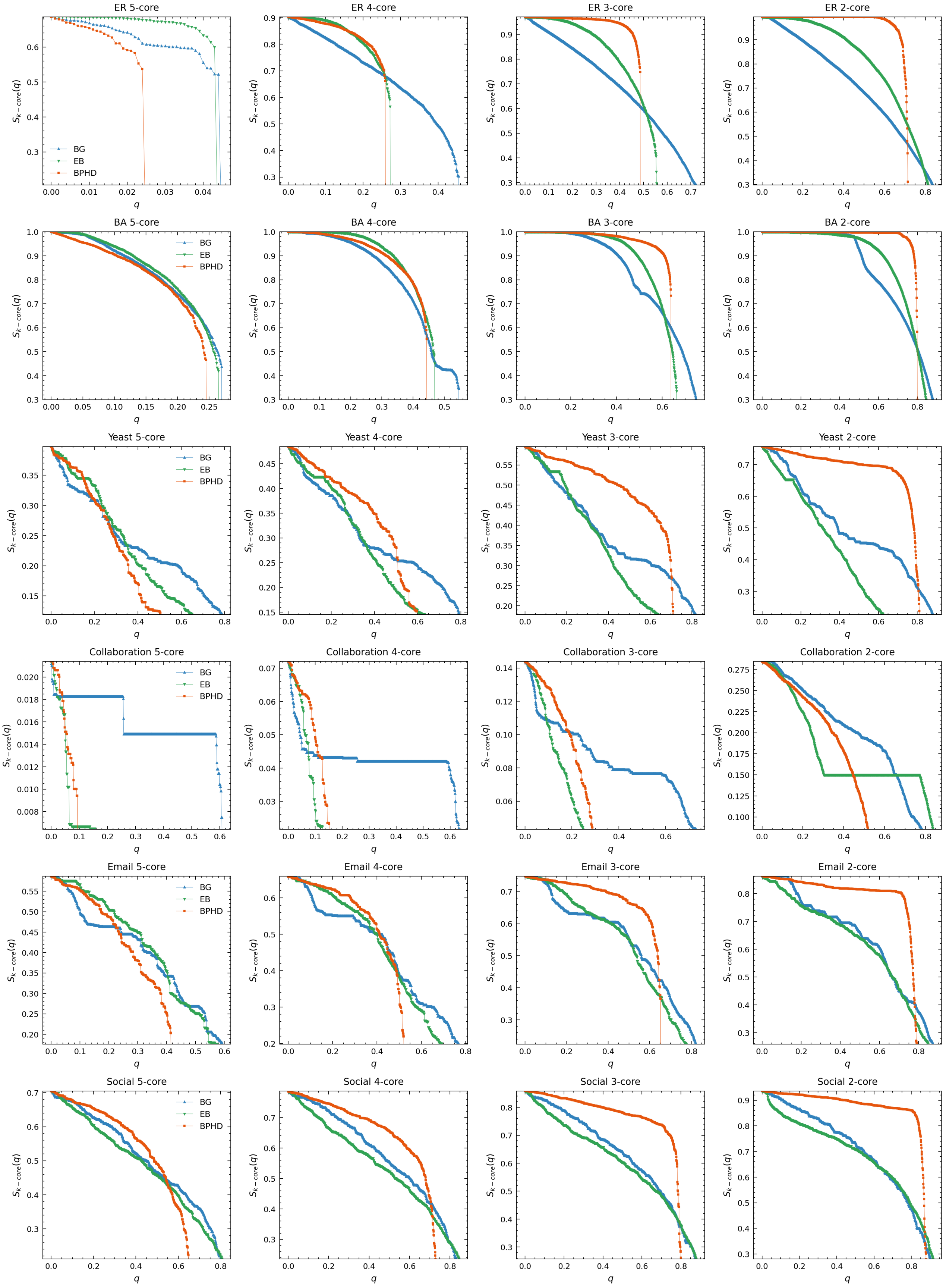

Figure S5: **Performance comparison of BPHD with two classical algorithms in higher-order structure dismantling.** The dismantling objectives are set as $H_k = 0.3H_k^0$, where $k = 5, 4, 3, 2$. H_k^0 represents the initial number of nodes within k-core.

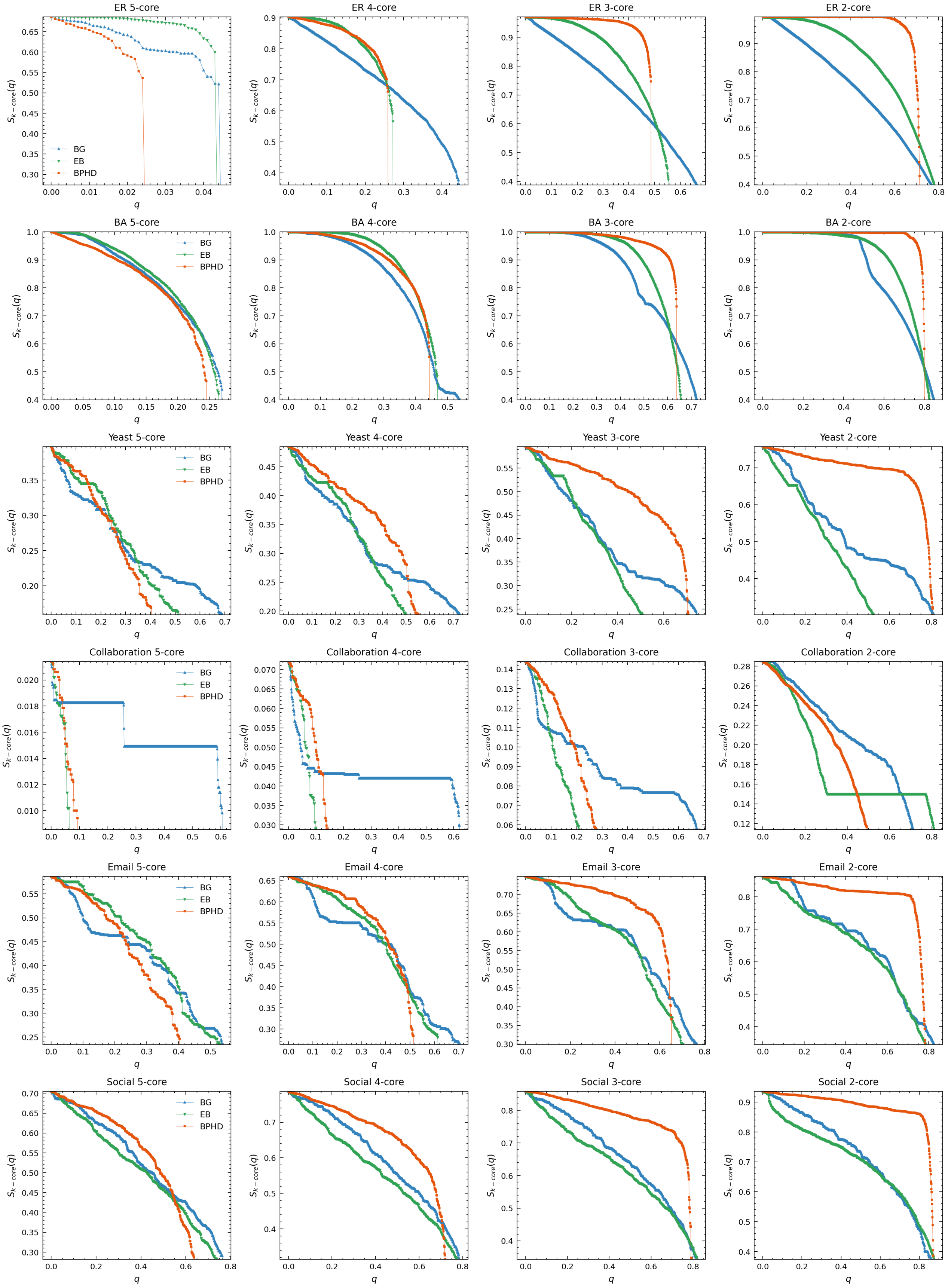

Figure S6: **Performance comparison of BPHD with two classical algorithms in higher-order structure dismantling.** The dismantling objectives are set as $H_k = 0.4H_k^0$, where $k = 5, 4, 3, 2$. H_k^0 represents the initial number of nodes within k-core.

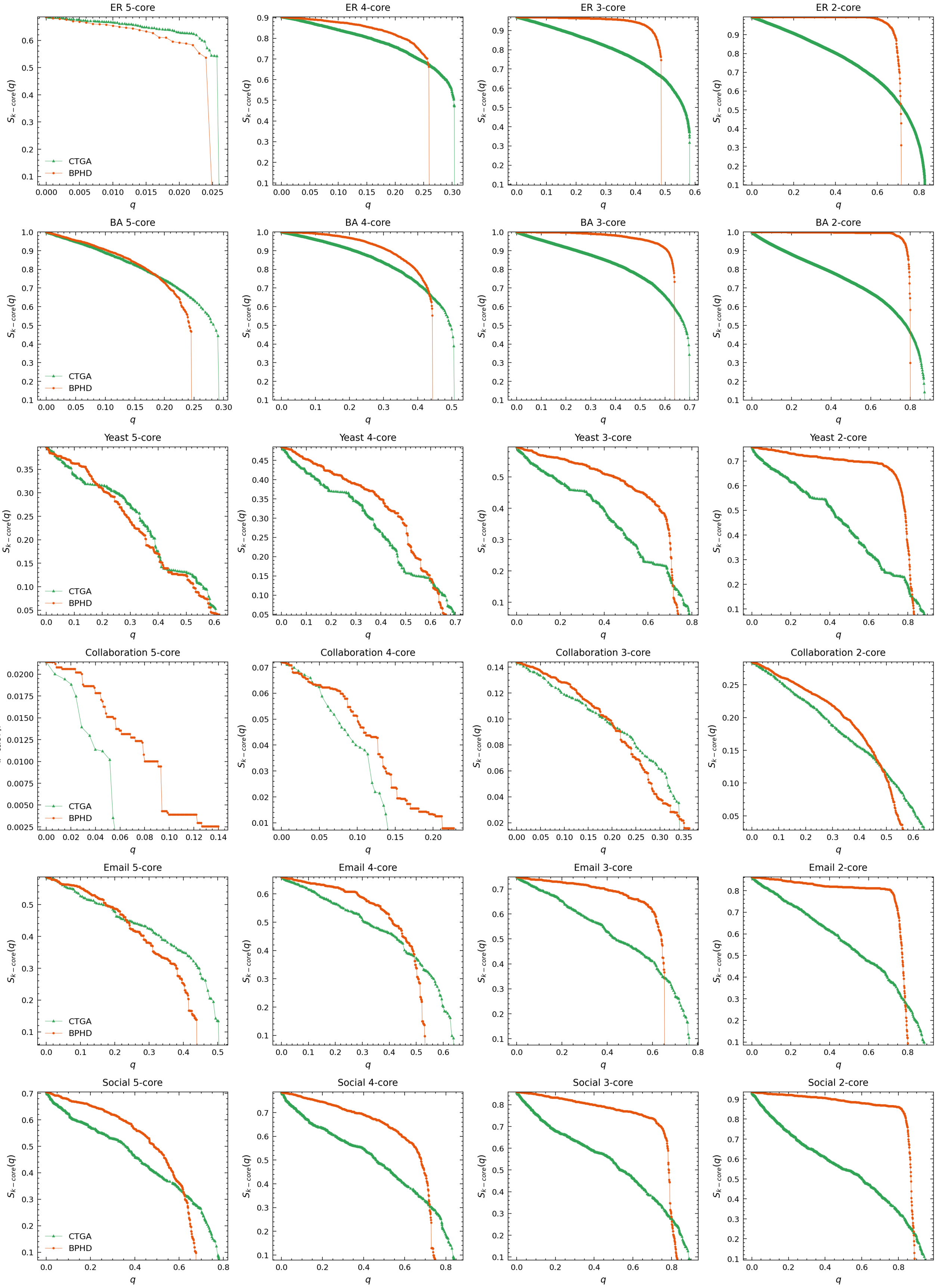

Figure S7: **Performance comparison of BPHD with the CTGA algorithm in higher-order structure dismantling.** The dismantling objectives are set as $H_k = 0.1H_k^0$, where $k = 5, 4, 3, 2$. H_k^0 represents the initial number of nodes within k-core.

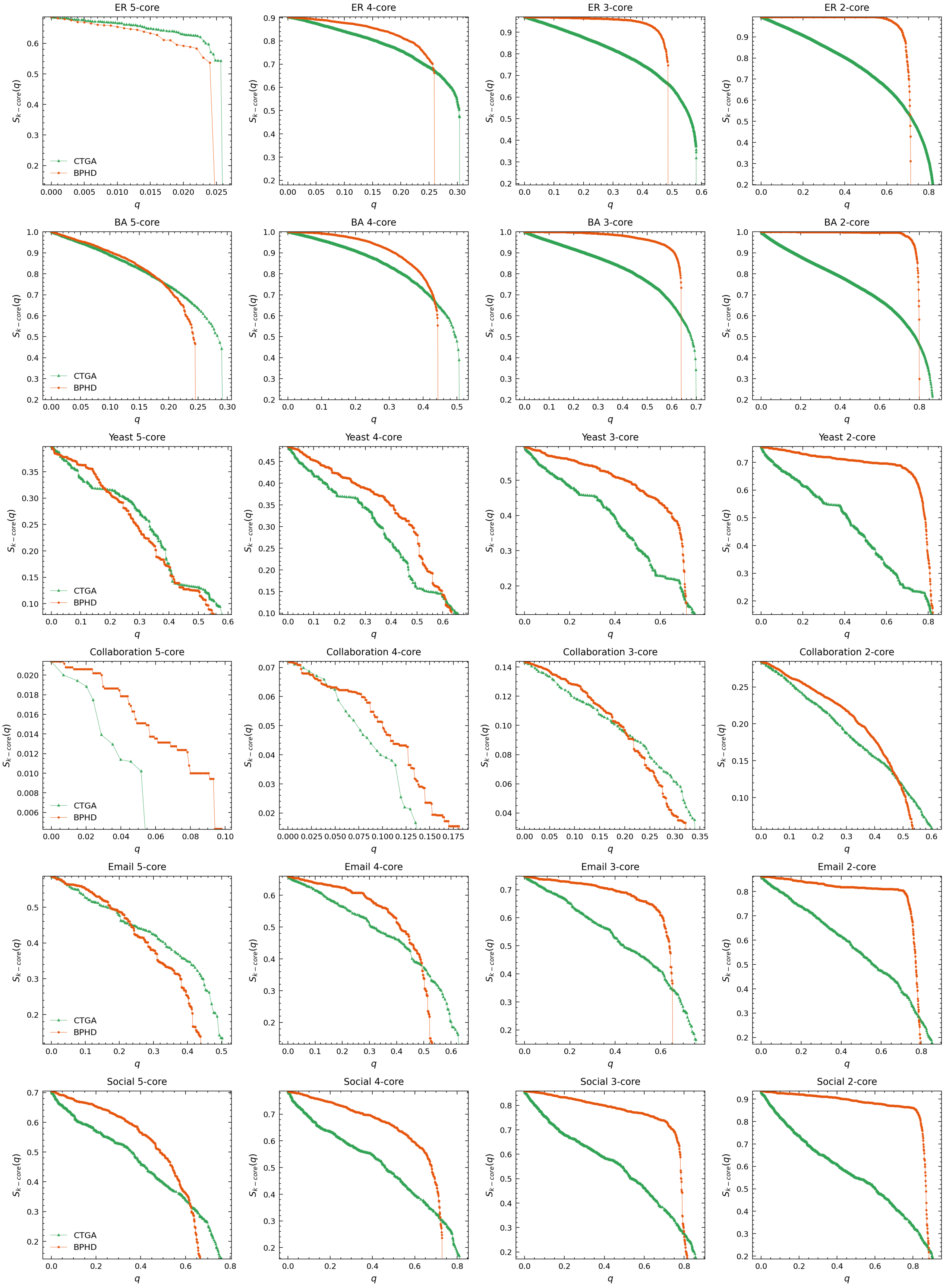

Figure S8: **Performance comparison of BPHD with the CTGA algorithm in higher-order structure dismantling.** The dismantling objectives are set as $H_k = 0.2H_k^0$, where $k = 5, 4, 3, 2$. H_k^0 represents the initial number of nodes within k-core.

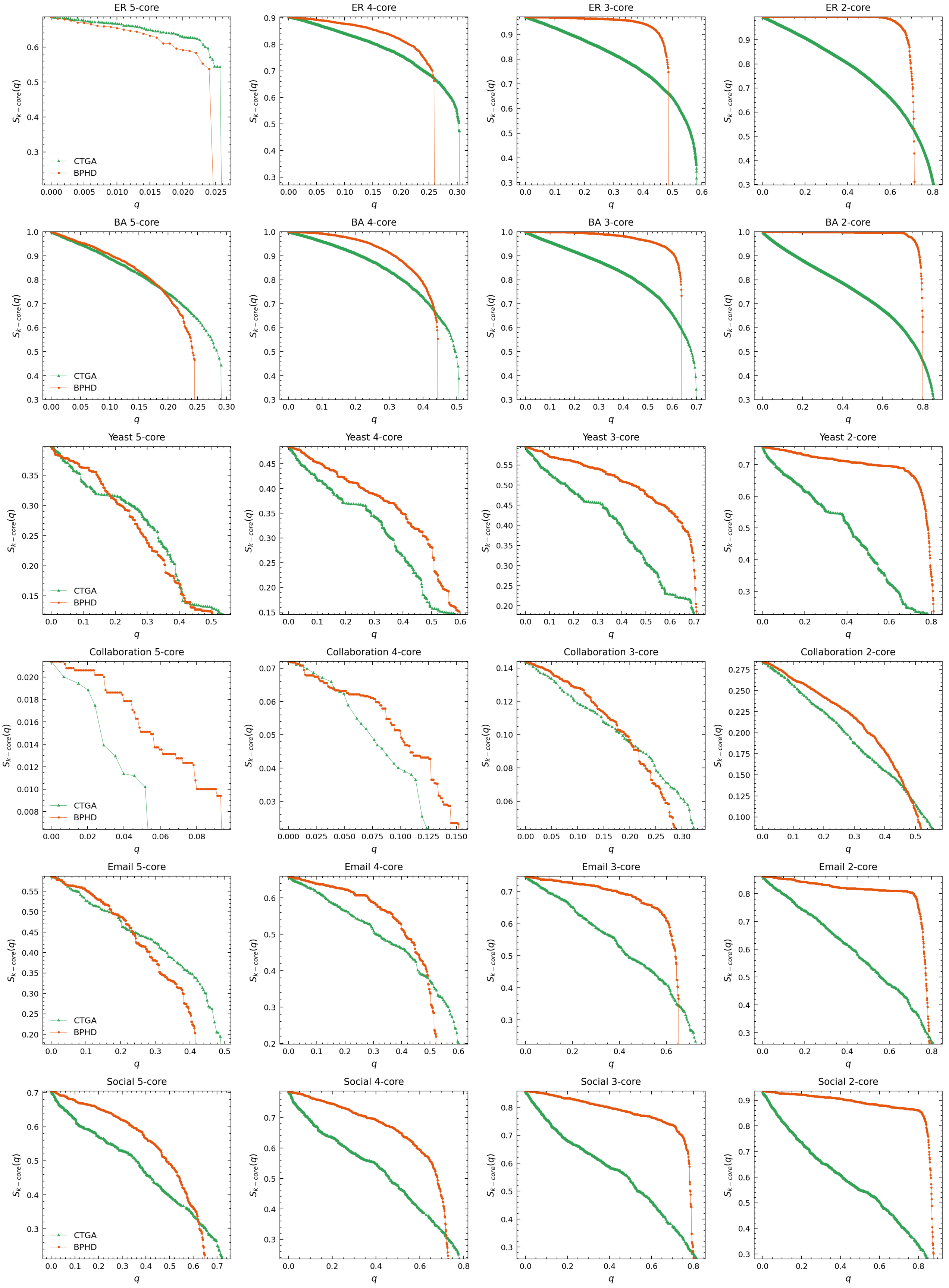

Figure S9: **Performance comparison of BPHD with the CTGA algorithm in higher-order structure dismantling.** The dismantling objectives are set as $H_k = 0.3H_k^0$, where $k = 5, 4, 3, 2$. H_k^0 represents the initial number of nodes within k-core.

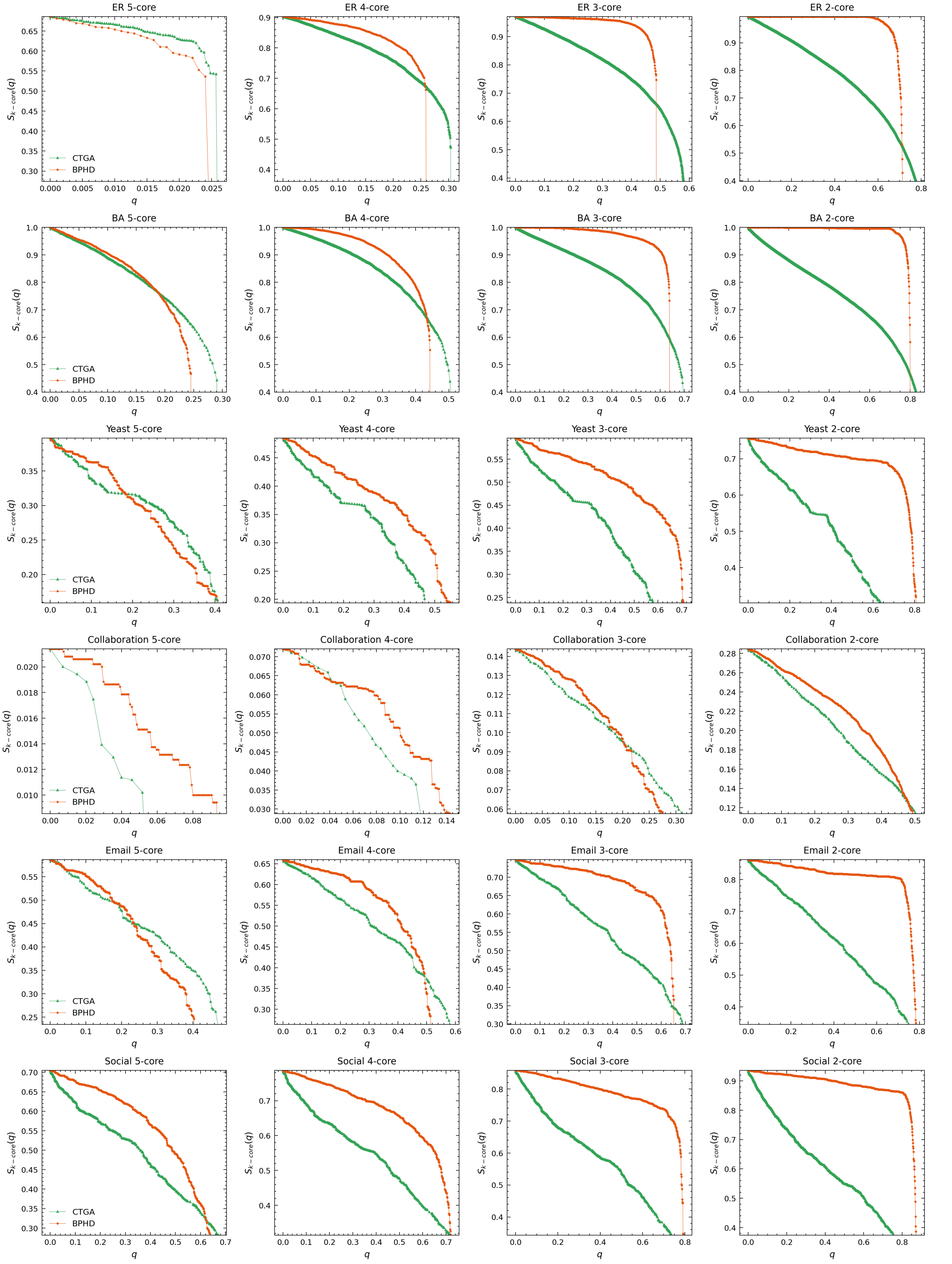

Figure S10: **Performance comparison of BPHD with the CTGA algorithm in higher-order structure dismantling.** The dismantling objectives are set as $H_k = 0.4H_k^0$, where $k = 5, 4, 3, 2$. H_k^0 represents the initial number of nodes within k-core.

2 Stochastic block model network with significant community structures

Here we use the stochastic block model [1] (SBM) to generate network with significant community structures that have a modularity of 0.814 when the network is divided according to the corresponding network blocks. The generated SBM network has 1,000 nodes which are divided into 100 blocks of size 10. The edge number of this SBM network is 2,742 , and the modularity is 0.814. We conducted the corresponding experiments in the generated SBM network. The dismantling results in SBM network are shown in Figure S11. The results show that the BPHD algorithm is not as effective as the baseline algorithm in connectivity dismantling, however, it performs better in dismantling higher-order structures of all orders. This is due to the fact that SBM networks have a large number of community structures. The baseline algorithm prioritises the dismantling of connections between communities, which leads to a rapid decrease in the size of the GCC, whereas the BPHD algorithm prioritises the dismantling of higher-order structures, which allows it to perform the connectivity dismantling task as well as the higher-order structure dismantling task at the same time.

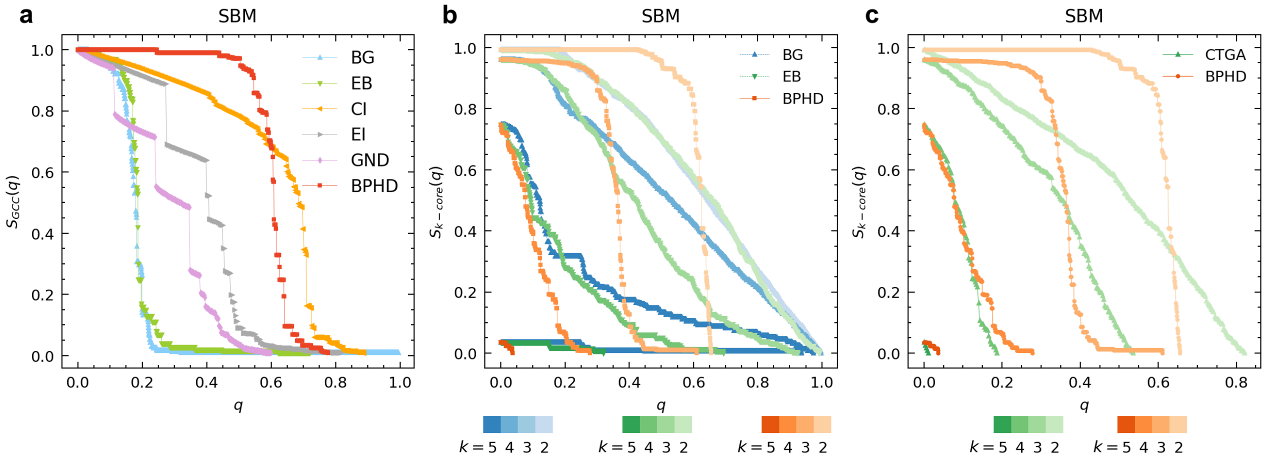

Figure S11: **Performance of BPHD in SBM model network.** Here, q and $S_{GCC}(q)$ represent the edge removal proportion and the corresponding relative size of GCC in the network, respectively. $S_{k-core}(q)$ represents the corresponding proportion of nodes with $k - core$ value equal to k in all branches. Each color corresponds to a specific algorithm, with shades from dark to light indicating the dismantling results for different orders k. The dismantling objectives are set as $C = 0.01N$ and $H = 0$.

References

[1] Paul W. Holland, Kathryn Blackmond Laskey, and Samuel Leinhardt. Stochastic blockmodels: First steps. *Social Networks*, 5(2):109–137, June 1983.
